# Interlaboratory Reproducibility of Standardized Hemagglutination Inhibition Assays

**DOI:** 10.1128/msphere.00953-21

**Published:** 2022-02-23

**Authors:** David C. Bibby, Michael Savanovic, Jinrong Zhang, Alessandro Torelli, Rienk E. Jeeninga, Luc Gagnon, Shannon L. Harris

**Affiliations:** a Seqirus, Inc., Cambridge, Massachusetts, USA; b VisMederi srl, Siena, Italy; c Viroclinics DDL, Rotterdam, The Netherlands; d Nexelis, Laval, Quebec, Canada; University of Maryland School of Medicine

**Keywords:** HI, influenza, precision, reproducibility, hemagglutination inhibition assay

## Abstract

The hemagglutination inhibition (HI) assay is a prominent and commonly accepted method used to determine quantitative antibody titers for influenza virus. However, the reproducibility and consistency of this assay may be affected by several factors, including its reliance on biological reagents that are difficult to standardize, such as red blood cells. This report assesses HI assay performance across three accredited, global laboratories when using test virus and a human serum panel aliquoted and distributed from a centrally located reagent stock. The panel of human sera comprised samples with expected low, medium, and high HI titers against two influenza viruses: A/H1N1/California/07/2009 and B/Victoria/Brisbane/60/2008. HI analysis followed a consensus test protocol. Overall, the HI assay reproducibility within each laboratory was high for both influenza strains, with a within-assay run and intraday precision of 100%. Interlab reproducibility was assessed by comparing the geometric mean titer (GMT) of each sample at each laboratory to the consensus GMT of the sample. A/H1N1 had 100% interlab reproducibility, and none of the individual laboratory GMT values exceeded a 2-fold difference compared to the consensus GMT in any tested sample. B/Victoria had an overall reproducibility of 83%. The results demonstrate that with standardization of key reagents and the use of a common protocol by trained staff, the biologically based HI assay can provide similar results between geographically dispersed laboratories.

**IMPORTANCE** Licensure of influenza vaccines relies on the hemagglutination inhibition (HI) assay as the primary method to determine quantitative functional antibody titers. The HI assay is also widely used for influenza virus surveillance, characterization, and epidemiology studies. However, the HI assay has a notable lack of reproducibility and consistency. If serology results are required from multiple concurrent studies supporting the development and regulatory approval of a product, the testing capacity of any given testing laboratory may be exceeded and data from more than one testing laboratory included in regulatory filings. Thus, understanding the reproducibility of HI assay results over time and between testing laboratories is necessary to support a robust clinical trial serology data set. Our results demonstrate that with standardization of key reagents and use of a common protocol by experienced and trained staff, the biologically based HI assay can provide similar results between geographically dispersed laboratories.

## INTRODUCTION

Licensure of influenza vaccines relies on the hemagglutination inhibition (HI) assay as the primary method to determine quantitative functional antibody titers. The HI assay is also widely used for influenza virus surveillance, characterization, and epidemiology studies (1, 2). The surface-expressed viral protein hemagglutinin (HA) interacts with sialic acid receptors on surfaces of cells, including red blood cells (RBCs) (3, 4). This interaction between influenza virus and RBCs results in hemagglutination. Antibodies directed against the HA disrupt or inhibit this interaction. Thus, the HI assay quantifies antibodies able to prevent virus-induced agglutination of RBCs. However, the HI assay has a notable lack of reproducibility and consistency, as shown by several interlaboratory studies, mainly because it requires reagents that are difficult to standardize, such as RBCs (5–8). Since influenza-related clinical and preclinical research groups collect and compare data from different test laboratories across the globe, there is an on-going need to compare results between laboratories. In 2011, the Consortium for the Standardization of Influenza Seroepidemiology (CONSISE) was formed to support standardization of assay performance at different test laboratories, and CONSISE members have collaborated on standardization of the HI assay (9).

The ability to compare and contrast immune responses to different influenza vaccines as measured by HI is another important scientific need. For a vaccine developer, clinical studies of the immunogenicity of vaccines occur over multiple influenza seasons. HI assays used in these studies are performed over extended periods of time, sometimes in more than one testing facility. Moreover, if serology results are required from multiple concurrent studies supporting the development and regulatory approval of a product, the testing capacity of any given testing laboratory may be exceeded and data from more than one testing laboratory included in regulatory filings. Thus, understanding the reproducibility of HI assay results over time and between testing laboratories is necessary to support a robust clinical trial serology data set. However, Zacour et al. have shown that use of a standardized operating procedure can produce highly reproducible results. In this study, laboratories repeatedly tested samples in HI assays using influenza A/H1N1 or A/H3N2 target viruses, and 95% to 100% of the samples tested met equivalence criteria (i.e., variation was within a single dilution [<2-fold]) with the all-laboratory consensus titers (6).

Here, we report on the analysis of a panel of human sera with expected low, medium, and high HI titers against two influenza viruses (A/H1N1/California/07/2009 and B/Victoria/Brisbane/60/2008) to determine if results tested at different locations can be reproduced.

## RESULTS

### Intralaboratory precision (within-assay run and intra- and interday).

As shown in Table 1, the within-assay run and intraday precision were both 100% over all three participating laboratories across both the A/H1N1 and B/Victoria HI assays. Chi-square tests were not performed, since there were no differences.

**TABLE 1:** Intralaboratory precision: precision within and across assays and days

Laboratory	Intra-assay precision,^*a*^ *n*/*N* (%)^*b*^			Intraday precision,^*c*^ *n*/*N* (%)			Interday precision,^*d*^ *n*/*N* (%)			Median intralab %GCV^*e*^ (min, max)	
	A/H1N1	B/Victoria	Both viruses	A/H1N1	B/Victoria	Both viruses	A/H1N1	B/Victoria	Both viruses	A/H1N1	B/Victoria
A	0/180 (100)	0/180 (100)	0/360 (100)	0/90 (100)	0/90 (100)	0/180 (100)	0/90 (100)	0/90 (100)	0/180 (100)	40.7 (0.0, 43.6)	22.2 (0.0, 43.6)
B	0/180 (100)	0/180 (100)	0/360 (100)	0/90 (100)	0/90 (100)	0/180 (100)	0/90 (100)	0/90 (100)	0/180 (100)	11.1 (0.0, 42.9)	0.0 (0.0, 43.6)
C	0/180 (100)	0/180 (100)	0/360 (100)	0/90 (100)	0/90 (100)	0/180 (100)	2/90 (97.8)	1/90 (98.8)	3/180 (98.3)	40.7 (22.2, 58.9)	40.7 (0.0, 58.9)
Overall	0/540 (100)	0/540 (100)	0/1080 (100)	0/270 (100)	0/270 (100)	0/540 (100)	2/270 (99.3)	1/270 (99.6)	3/540 (99.4)	40.7 (0.0, 58.9)	22.2 (0.0, 58.9)

a Number of samples with replicate ratios differing by more than twofold per total number of replicate ratios.

b Percent precision, calculated as (1 − [*n*/*N*]) × 100.

c Number of samples with GMT ratios for assays performed within a day differing by more than 2-fold per total number of GMT ratios.

d Number of samples with GMT ratios for pairwise comparison between days differing by more than 2-fold per total number of pairwise comparisons between days (e.g., day 1-day 2, day 1-day 3, day 2-day 3).

e Interday variability is expressed as the percent geometric coefficient of variation, %GCV = 100(exp(*s*) − 1, where *s* is the standard deviation of the natural logarithm of the titers of the geometric mean titers combined.

Interday precision for Lab A and B for both assays was 100% (Table 1). In Lab C, however, the difference in the interday sample GMT ratio in the A/H1N1 assay for two samples was >2-fold (97.8% precision), and the difference was >2-fold for one sample in the B/Victoria assay (98.9% precision). Across the two strains, Lab C had 98.3% interday precision.

Overall, across the three laboratories, interday precision was 99.3% for A/H1N1 and 99.6% for B/Victoria. Across both assays for the three laboratories, the interday precision was 99.4%. A chi-square test suggests no evidence of differences between laboratories (*P = *0.107, Fischer’s exact test).

As shown in Table 1, the median percent geometric coefficient of variation (%GCV) at Lab A was 40.7% (minimum, 0.0%; maximum, 43.6%) for A/H1N1 and 22.2% (0.0%, 43.6%) for B/Victoria. At Lab B, median %GCV was 11.1% (0.0%, 42.9%) for A/H1N1 and 0.0% (0.0%, 43.6%) for B/Victoria. At Lab C, the median %GCV was 40.7% (22.2%, 58.9%) for A/H1N1 and 40.7% (0.0%, 58.9%) for B/Victoria. Overall variability for the two assays across three laboratories was modest, with a median %GCV of 40.7% (0.0%, 58.9%) for A/H1N1 and 22.2% (0.0%, 58.9%) for B/Victoria.

### Interlaboratory precision.

Reproducibility of A/H1N1 was 100% in all three laboratories. In Lab C, reproducibility of B/Victoria was also 100%. In Lab B, the difference was >2-fold for six samples of B/Victoria, for 80% reproducibility. Reproducibility of B/Victoria in Lab A, where the difference was >2-fold for nine samples, was 70%. Overall, the difference exceeded 2-fold for 15 samples across all laboratories, for 83% reproducibility (Table 2). Chi-square testing suggests evidence of differences between laboratories (χ^2^ = 10.08; *P =* 0.006).

**TABLE 2:** Interlaboratory precision expressed as number of samples for which lab GMT to consensus GMT ratios differed by >2-fold per total number of GMT ratios^*a*^

Laboratory	A/H1N1, *n*/*N* (%)^*b*^	B/Victoria, *n*/*N* (%)
A	0/30 (100)	9/30 (70)
B	0/30 (100)	6/30 (80)
C	0/30 (100)	0/30 (100)
Overall	0/90 (100)	15/90 (83)

a A consensus GMT for each sample was calculated across all replicates in all labs. The consensus GMT was then compared to the GMT determined at each lab for each sample, and fold differences were calculated.

b Percentages are percent precision, calculated as (1 − [*n*/*N*]) × 100.

**FIG 1:**
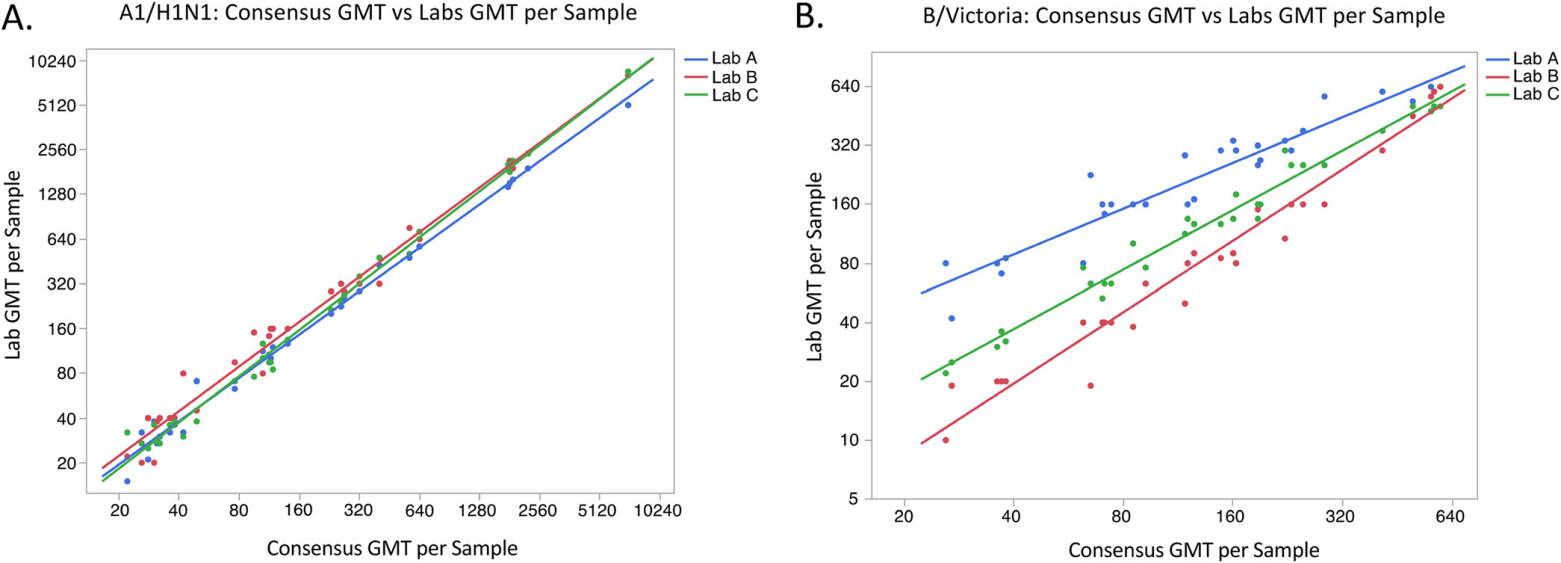
Scatterplots (with lines of best fit) of GMT of each sample from each laboratory compared to the consensus GMT of the sample for the hemagglutination inhibition (HI) value using A/H1N1 (A) or B/Victoria (B).

The median %GCV of A/H1N1 was 41.9% (minimum, 21.4%; maximum, 70.5%) and for B/Victoria was 68.4% (24.7%, 192%) (see Tables S1 and S2 in the supplemental material). To visualize variability for each assay, scatterplots were created for the GMT of each sample for each laboratory plotted against the consensus GMT of the sample. As shown in Fig. 1, there was little variability between the laboratories for the A/H1N1 assay, while variability was greater for the B/Victoria assay, with the three laboratories having three distinct groupings of data points.

10.1128/msphere.00953-21.1 TABLE S1 Laboratory and consensus GMT and %GCV for A/H1N1 panel sera. Download Table S1, DOCX file, 0.02 MB. Copyright © 2022 Bibby et al. 2022 Bibby et al. https://creativecommons.org/licenses/by/4.0/ This content is distributed under the terms of the Creative Commons Attribution 4.0 International license.

10.1128/msphere.00953-21.2 TABLE S2 Laboratory and consensus GMT and %GCV for B/Victoria panel sera. Download Table S2, DOCX file, 0.02 MB. Copyright © 2022 Bibby et al. 2022 Bibby et al. https://creativecommons.org/licenses/by/4.0/ This content is distributed under the terms of the Creative Commons Attribution 4.0 International license.

### Interlaboratory trending and correlations.

**FIG 2:**
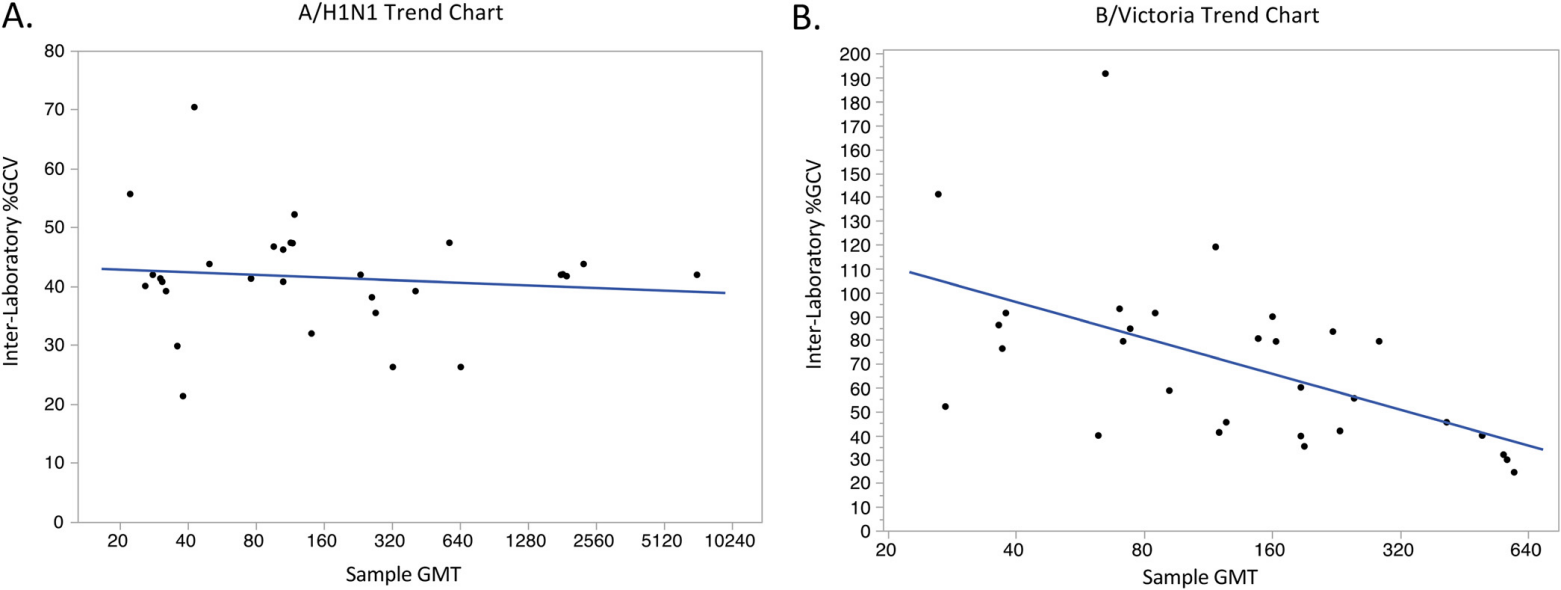
A/H1N1 (A) and B/Victoria (B) interlab %GCV trends across the range of titers obtained. The interlab GCV for each sample was plotted against the consensus GMT for each sample for the hemagglutination inhibition (HI) value using A/H1N1 or B/Victoria.

Trend charts were constructed for both assays to examine trending of the variability of the assays across the range of titers obtained. The interlab %GCV for each sample was plotted against the consensus GMT for each sample. Assay variability for A/H1N1 was consistent across the range of titers obtained, whereas the variability of the B/Victoria assay is high at the low end of the titer range and decreases with increasing GMT until variability becomes comparable to the assay variability observed in the A/H1N1 assay (Fig. 2).

Correlation analysis was performed on the raw data titer values for each assay to determine the Pearson correlation coefficient between laboratories (Table 3). For A/H1N1, all correlations between laboratories were significant, with *r* values between 0.92 and 0.94 (*P < *0.0001). For B/Victoria, strongly positive relationships were observed between each laboratory pairing: Lab A and B, *r *= 0.80 (*P < *0.0001); Lab A and C, *r *= 0.77 (*P < *0.0001); Lab B and C, *r *= 0.82 (*P < *0.0001).

**TABLE 3:** Pearson correlation coefficient between laboratories^*a*^

Precision coefficient (95% CI)	A/H1N1	B/Victoria
Lab A, Lab B	0.93 (0.92–0.95)	0.80 (0.76–0.84)
Lab A, Lab C	0.92 (0.91–0.94)	0.77 (0.73–0.81)
Lab B, Lab C	0.94 (0.93–0.95)	0.82 (0.78–0.85)

a Precision coefficient is equivalent to the Pearson correlation coefficient, a measure of deviation from the best fit line.

### HI B/Victoria lot-to-lot comparison.

No differences were >2-fold for both within-assay runs (0/54 for both lots) and interday (0/54 for both lots) precision, for 100% precision. The median %GCV was 43% (minimum, 0.0%; maximum, 46.2%) for lot 1B and 32.7% (0.0%, 46.2%) for lot 2B (Table 4).

**TABLE 4:** B/Victoria lot to lot intra-assay results

Intra-assay precision,^*a*^ *n*/*N* (%)^*b*^		Interday precision,^*c*^ *n*/*N* (%)^*b*^		Median intralot %GCV^*d*^ (min, max)	
Lot 1B	Lot 2B	Lot 1B	Lot 2B	Lot 1B	Lot 2B
0/54 (100)	0/54 (100)	0/54 (100)	0/54 (100)	43.0 (0.0, 46.2)	32.7 (0.0, 46.2)

a Number of samples with replicate ratios differing by more than twofold/total number of replicate ratios.

b Percent precision, calculated as (1 − [*n*/*N*]) × 100.

c Number of samples with GMT ratios for pairwise comparison between days differing by more than 2-fold/total number of pairwise comparisons between days (e.g., day 1-day 2, day 1-day 3, day 2-day 3).

d Interday variability is expressed as the geometric coefficient of variation, %GCV = 100(exp(*s*) − 1, where *s* is the standard deviation of the natural logarithm of the titers of the geometric mean titers combined.

Likewise, no differences in the interlot comparison (the comparison of each lot’s GMT per sample) were >2-fold (0/18), for 100% repeatability. The median %GCV calculated across all replicates for both lots and all samples was 40.7% (0.0%, 49.2%).

Analysis of variance (ANOVA) of the observed GMTs (168.4 for lot 1B and 220.5 for lot 2B) suggests evidence of differences in the overall observed GMT between the two virus lots (*F*_1, 214_ = 4.2808; *P = *0.0397).

## DISCUSSION

Like Zacour et al. (6), we demonstrated that HI results can be reproduced between different testing laboratories using a standardized assay protocol and shared key critical reagents. To minimize the HI assay variability, all three laboratories contributed to an agreed-upon consensus assay protocol. The virus stocks and serum panel in the study were prepared at Seqirus, and the aliquots were shipped to each laboratory. Receptor-destroying enzyme (RDE) and serum diluent were from the same manufacturer but ordered separately at each laboratory. Since the HI assay requires fresh turkey red blood cells (TRBCs), each laboratory sourced their own lots or obtained TRBCs from local suppliers. The use of independently obtained assay buffers, RDE, and RBCs would be expected in a real-world setting for testing of samples across multiple locations.

Overall, the HI assay reproducibility within each laboratory was high for both assays (A/H1N1 and B/Victoria), with the within-assay run and intraday precision being 100% for both assays. Interday precision had just three occurrences of greater than 2-fold differences, two for A/H1N1 (99.3% precision) and one for B/Victoria (99.6% precision), which occurred at the same laboratory. Overall, across both assays, precision was 99.4%. Variability within laboratories was low, with a median %GCV of 40.7% (minimum, 0.0%; maximum, 58.9%) for A/H1N1 and 22.2% (0.0%, 58.9%) for B/Victoria. Interlab reproducibility was 100% for A/H1N1, and none of the differences between the GMT values obtained at each laboratory and the consensus GMT calculated from all three laboratories exceeded 2-fold. The overall 83% reproducibility for B/Victoria resulted from 15 differences that were >2-fold at Labs A (reproducibility 70%) and B (80%); the reproducibility of B/Victoria was 100% at Lab C. Reflecting these findings, the overall interlab variability for A/H1N1 was low (%GCV, 41.9% [21.4%, 70.5%]), whereas for B/Victoria variability was higher (%GCV, 68.4% [24.7%, 192%]). Thus, with a reasonable amount of standardization (i.e., shared assay protocol, shared viral lots, and shared test samples), there was good agreement both within and between the laboratories that participated in this study.

The FLUCOP collaborative (Waldock et al.) recently reported similar reductions in interlaboratory variation through the use of a consensus protocol and common critical reagents (10). This group also demonstrated that the use of a pooled postvaccinated human serum pool as a reference standard provided similar reductions in interlaboratory variation when different protocols and reagents are used. The reference standard that provided the greatest reduction in interlaboratory variation was created from volunteers exposed to the virus strains that most closely matched the virus targets used in the HI assays. The authors suggest that the reference standards from pooled vaccinated individuals could be used for at least 5 years. However, large shifts in the circulating virus reduce the effectiveness if there is a large mismatch between the virus used to create the reference standard and the virus target being evaluated. The work of Carreño et al. to develop a reference standard containing anti-stalk antibodies to aid in harmonizing stalk-based immunogenicity assays supports the importance of using a reference standard that is related to the antibody population to be measured (11).

Interestingly, Waldock et al. demonstrated no cumulative reduction in interlaboratory variation when using a consensus protocol, common critical reagents, and a reference standard (10). They suggest that the improvements in assay performance obtained either through the use of a consensus protocol and common reagents or the use of a reference standard, are close to the limits of improvement possible. The remaining variation may be inherent to the existing assay format and its reliance on TRBC as one example of interlaboratory variation that cannot be easily overcome. The TRBC used in our study may account for some of the observed differences in the results obtained from each laboratory. Variations in local handling practices and food sources between Europe and North America could lead to TRBC with slightly different performance characteristics. A key source of variation in HI for B/Victoria was virus lot provided to the laboratories. Since the original lot of B/Victoria was insufficient for use at all three labs, Lab A used a different lot from that used by Labs B and C. To address this issue, the two B/Victoria virus lots were tested side by side, and we did find differences in the medium- and high-titer groups using one-way ANOVA. These findings could explain some of the variations in the interlab HI assay comparison for the B/Victoria strain. However, this was only one of several variables assessed in this study, and the overall reproducibility of the B/Victoria assay remained high, at 83%.

Evidence from all laboratories suggests that A/H1N1 had more stable hemagglutination in the HI assay than the B/Victoria virus strain in this study. The B/Victoria strain had a more diffused hemagglutination, particularly with the lower titer samples, which may have contributed to a more subjective interpretation of agglutination. Each of the laboratories determined the endpoint titer using a standard manual tilt method. Such manual readouts are, in general, difficult to standardize across laboratories based upon the experience of the operators performing the assays. Some instrumentation manufacturers have introduced automated instruments for the imaging of HI assays that allow for a higher throughput and more standardized determination of the HI titer, including the CypherOne analyzer from Indevr and the HIVE T670 imager from Sanofi Pasteur VaxDesign (8, 9). The inclusion of such automated readouts could further reduce assay variability between different testing laboratories by reducing the subjective interpretation of HI titers.

Overall, the within-laboratory HI assay reproducibility was high for both A/H1N1 and B/Victoria assays, with within-assay run and intraday precision of 100% for both assays. Between-laboratory reproducibility was 100% for A/H1N1 and 83% for B/Victoria. These results demonstrate that with standardization of key reagents and use of a common protocol by experienced and trained staff, the biologically based HI assay can provide similar results between geographically dispersed laboratories.

## MATERIALS AND METHODS

### Study design.

The human serum panels were tested using the HI assay at three testing laboratories, VisMederi srl (Siena, Italy), Viroclinics DDL (Rotterdam, The Netherlands), and Nexelis (Laval, Canada), which are all members of the Coalition for Epidemic Preparedness Innovations (CEPI) global laboratory network. Each testing laboratory was assigned a code letter: A, B, or C. Human sera used in the analyses were from a previously completed influenza vaccine study (ClinicalTrials registration no. NCT02214225), wherein subjects had been administered an influenza vaccine from the 2014–2015 Northern Hemisphere influenza season. That study was conducted in compliance with the Declaration of Helsinki, International Conference of Harmonization–Good Clinical Practice and applicable laws and regulations, and all participants provided written informed consent (12).

Serological analysis followed a consensus test protocol that was provided by the sponsor and was developed based on protocols used in the participating laboratories as well as the World Health Organization. At each participating laboratory, the human serum panels were tested in two replicates in each of six assays performed on three separate days, two assays per day for each target virus. Samples were tested at the testing laboratories using the nested design outlined in Table 5. In each testing laboratory, one experienced analyst was responsible for all tests, for a total of three analysts across the three testing laboratories. The serum panel and viral reagents were provided by the sponsor; each laboratory used their own ancillary assay reagents, material, and equipment.

**TABLE 5:** Experimental design for interlaboratory comparison

Lab A/analyst A			Lab B/analyst B			Lab C/analyst C		
Day 1	Day 2	Day 3	Day 1	Day 2	Day 3	Day 1	Day 2	Day 3
Run 1	Run 3	Run 5	Run 1	Run 3	Run 5	Run 1	Run 3	Run 5
Run 2	Run 4	Run 6	Run 2	Run 4	Run 6	Run 2	Run 4	Run 6

### Serum samples.

Based on the reported HI titer from the original trial, available serum volume, and informed consent from participants (12), two panels of sera, each containing 30 postvaccination sera, were created. Each panel contained approximately 10 sera with low (≤40), medium (between 80 and 320), and high (≥640) HI titers against either A/H1N1/California/07/2009 or B/Victoria/Brisbane/60/2008. The panel sera were aliquoted and stored at the sponsor site until they were shipped for use to the laboratories involved. Samples were shipped on dry ice and stored at −80°C until required.

### Influenza viruses.

Seqirus produced and aliquoted lot 1A of cell-based influenza virus A/H1N1/California/07/2009 and lots 1B and 2B of cell-based influenza virus B/Victoria/Brisbane/60/2008. The viral reagents were shipped on dry ice and stored at the participating laboratories at −80°C until required. A new aliquot of virus was thawed for use in each assay.

There was an insufficient volume of B/Victoria strain lot 1B for all three laboratory HI assay tests, so Lab A received lot 2B for the initial interlab HI assay comparison, whereas Labs B and C received lot 1B. The two lots of B/Victoria were then compared at Lab A to test for differences, in separate assays, subsequent to the interlab HI assay comparison.

### Other key reagents.

Each testing laboratory acquired key reagents for the HI assay, such as the positive- and negative-control sera, receptor-destroying enzyme (RDE), and turkey red blood cells (TRBC) for themselves, and these reagents were not controlled across the three testing laboratories. RDE was prepared according to the manufacturer’s instructions at each laboratory.

### HI assay.

To remove nonspecific inhibitors of HA, sera were incubated with RDE at a ratio of 1:5 serum-RDE dilution for 16 to 18 h at 37°C, followed by inactivation of the RDE at 56°C for 60 min. Immediately after heat inactivation, RDE-treated sera were preabsorbed on TRBC by gently mixing the sera with a solution of 15% TRBC in a 1:1 ratio and incubating for 30 min at room temperature. TRBC was removed by centrifugation, resulting in RDE-treated, TRBC-absorbed serum samples at a dilution of 1:10. The viral reagents were diluted for use at 4 hemagglutinating units (HU) in 25 μL. To perform the HI assay, pretreated sera were serially 2-fold diluted from the initial 1:10 dilution, mixed with the diluted virus 1:1 (25 μL diluted sera and 25 μL diluted virus), and incubated at room temperature for 60 min. Next, 50 μL of 0.5% TRBC was added, followed by incubation at room temperature for 60 min. Assay plates were tilted to read, and the titer was reported as the reciprocal of the highest serum dilution in which agglutination was completely inhibited.

Human serum panel samples were tested in two replicates in each of six assays. Therefore, up to 12 titers were generated for each sample at each testing laboratory for each target virus. The standard positive- and negative-control sera used by each testing laboratory were included in each assay and used to determine if an assay was acceptable based upon the testing laboratory's standard assay review process. The data from the positive and negative controls were not included in the report.

### HI B/Victoria lot-to-lot comparison.

The lot-to-lot comparison used a subset of the human serum panel to compare the two lots of B/Victoria strains at Lab A only. Fresh aliquots of 18 human sera from a previous serum panel (6 sera randomly chosen from each titer group that consisted of low, medium, and high titers) and the two B/Victoria virus lots were tested using the HI assay for B/Victoria at Lab A. One analyst performed one run per day for each B/Victoria virus lot. Each run consisted of duplicate titers across 3 days of 18 samples, for a total of 108 data points for each lot.

### Statistical analysis.

Titers were determined as the reciprocal of the highest serum dilution in which there is complete inhibition of the hemagglutination. There was no interpolating of results between serum dilutions. Titers of <10 (below the first measurable titer) were assigned a value of 5, one-half the lower limit of the assay, for calculations. A chi-square test of association (χ^2^) was used to assess the significance of differences in all comparisons. The Fishers exact test rather than the asymptotic test was used to accommodate expected counts of less than 5.

### (i) Within-assay precision.

Within-assay run precision was assessed as the ratio of replicates for each sample within an assay, within each run, and the proportion of samples with a replicate ratio greater than a 2-fold difference.

### (ii) Intraday precision.

The geometric mean titer (GMT) for a sample within an assay was calculated using the replicate titers of each run, within each day. The intraday assay precision was assessed as the ratio of GMTs for each sample obtained within a day and the proportion of samples with a GMT ratio greater than 2-fold.

### (iii) Interday precision.

The GMT for a sample for each day was calculated using all replicate titers obtained across the two assays performed in a day. The interday precision was assessed as the ratio of the GMTs for each sample from each pairwise comparison across days (e.g., GMT from day 1 to GMT from day 2; GMT from day 1 to GMT from day 3; and GMT from day 2 to GMT from day 3) and the proportion of samples with a GMT ratio greater than 2-fold. The percent geometric coefficient of variation (%GCV) measured variability between the endpoint titers and was calculated for each lab, for each assay, and across all replicates and was determined using the formula 100[exp(*s*) − 1], where *s* is the standard deviation of the natural logarithm of the titers of the geometric mean titers combined.

### (iv) Interlab precision.

The interlab precision was assessed by comparing the GMT calculated across all replicates for each sample from each laboratory to the consensus GMT, which was calculated as the GMT of all replicates for each sample for all laboratories to assess any 2-fold differences. The magnitude of titer variability was quantified by calculating the %GCV of all sample titers for all laboratories combined. The GMTs of the titers obtained from each laboratory were graphed against the consensus GMT, and the interlab correlations between the titers were assessed utilizing the Pearson correlation coefficient, which is equivalent to the precision coefficient and a measure of the deviation from the best fit line.

### (v) B/Victoria lot-to-lot comparison.

Intra-assay analysis of the 108 data points for each B/Victoria virus lot consisted of comparisons of replicates within each day and calculation of any 2-fold differences for each sample. The GMT of each day’s replicates was determined for each sample; 2-fold differences were calculated; and the variability of each lot was determined by the calculation of the %GCV for each sample within each lot. Interlot comparison consisted of comparing each sample’s overall GMT for each lot and calculation of any differences of >2-fold. The %GCV was calculated across all replicates for both lots and all samples.

A one-way ANOVA was performed on the overall, log-transformed data to identify any evidence of differences between observed GMTs for lots 1B and 2B. In addition, samples were separated into three separate titer range groups (low, medium, and high GMT) to limit any undue influence of one group on the other. ANOVA was performed for each group between the two B/Victoria virus lots using log-transformed data.

Following transfer of data, HI assay precision was evaluated with interassay, intraday, interday, and interlab comparisons by a Seqirus biostatistician using JMP v15.1.0 (SAS, Cary, North Carolina).
