# Supplementary material for: Interlaboratory Reproducibility of Standardized Hemagglutination Inhibition Assays
Source: mSphere. 2022 Feb 23;7(1):e00953-21. doi: 10.1128/msphere.00953-21 (PMC8865924; doi:10.1128/msphere.00953-21)
Supplement: TABLE S1 [file msphere.00953-21-st001.docx]

**TABLE S1** Laboratory and consensus geometric mean titers (GMT) and percentage geometric coefficient of variation (%GCV) for A/H1N1 panel sera

| **Sample ID** | **Consensus GMT*^a^*** | **Lab A**  **GMT*^b^*** | **Lab B**  **GMT*^b^*** | **Lab C**  **GMT*^b^*** | **%GCV between 3 labs** |
| --- | --- | --- | --- | --- | --- |
| H1N1-1 | 49 | 71 | 45 | 38 | 43.9 |
| H1N1-2 | 22 | 15 | 22 | 32 | 55.8 |
| H1N1-3 | 31 | 27 | 38 | 28 | 40.9 |
| H1N1-4 | 28 | 21 | 40 | 25 | 42.0 |
| H1N1-5 | 32 | 30 | 40 | 27 | 39.3 |
| H1N1-6 | 30 | 38 | 20 | 36 | 41.4 |
| H1N1-7 | 26 | 32 | 20 | 27 | 40.2 |
| H1N1-8 | 38 | 36 | 40 | 38 | 21.4 |
| H1N1-9 | 42 | 32 | 80 | 30 | 70.5 |
| H1N1-10 | 36 | 32 | 40 | 36 | 30.0 |
| H1N1-11 | 118 | 120 | 160 | 85 | 52.3 |
| H1N1-12 | 115 | 101 | 160 | 95 | 47.4 |
| H1N1-13 | 105 | 113 | 101 | 101 | 46.3 |
| H1N1-14 | 113 | 107 | 143 | 95 | 47.5 |
| H1N1-15 | 76 | 63 | 95 | 71 | 41.4 |
| H1N1-16 | 95 | 76 | 151 | 76 | 46.8 |
| H1N1-17 | 259 | 226 | 320 | 240 | 38.2 |
| H1N1-18 | 231 | 202 | 285 | 214 | 42.0 |
| H1N1-19 | 105 | 113 | 80 | 127 | 40.9 |
| H1N1-20 | 140 | 127 | 160 | 135 | 32.1 |
| H1N1-21 | 403 | 427 | 320 | 479 | 39.3 |
| H1N1-22 | 269 | 254 | 285 | 269 | 35.6 |
| H1N1-23 | 320 | 285 | 320 | 359 | 26.4 |
| H1N1-24 | 640 | 570 | 640 | 718 | 26.4 |
| H1N1-25 | 570 | 479 | 761 | 508 | 47.5 |
| H1N1-26 | 1810 | 1522 | 2153 | 1810 | 42.1 |
| H1N1-27 | 1881 | 1613 | 1918 | 2153 | 41.8 |
| H1N1-28 | 1776 | 1437 | 1918 | 2032 | 42.0 |
| H1N1-29 | 2237 | 1918 | 2416 | 2416 | 43.9 |
| H1N1-30 | 7103 | 5120 | 8127 | 8611 | 42.0 |

*^a^*Consensus GMT for each sample was calculated across all replicates, of all laboratories.

*^b^*Laboratory GMT for each sample was calculated across all replicates within a laboratory.
