# Supplementary material for: Interlaboratory Reproducibility of Standardized Hemagglutination Inhibition Assays
Source: mSphere. 2022 Feb 23;7(1):e00953-21. doi: 10.1128/msphere.00953-21 (PMC8865924; doi:10.1128/msphere.00953-21)
Supplement: TABLE S2 [file msphere.00953-21-st002.docx]

**TABLE S2** Laboratory and consensus geometric mean titers (GMT) and percentage geometric coefficient of variation (%GCV) for B/Victoria panel sera

| **Sample ID** | **Consensus GMT*^a^*** | **Lab A GMT*^b^*** | **Lab B GMT*^b^*** | **Lab C GMT*^b^*** | **%GCV between 3 labs** |
| --- | --- | --- | --- | --- | --- |
| BVic-1 | 26 | 80 | 10 | 22 | 141.4 |
| BVic-2 | 27 | 42 | 19 | 25 | 52.3 |
| BVic-3 | 92 | 160 | 63 | 76 | 58.9 |
| BVic-4 | 85 | 160 | 38 | 101 | 91.5 |
| BVic-5 | 65 | 226 | 19 | 63 | 192.0 |
| BVic-6 | 38 | 85 | 20 | 32 | 91.5 |
| BVic-7 | 160 | 339 | 90 | 135 | 90.0 |
| BVic-8 | 36 | 80 | 20 | 30 | 86.5 |
| BVic-9 | 62 | 80 | 40 | 76 | 40.2 |
| BVic-10 | 37 | 71 | 20 | 36 | 76.6 |
| BVic-11 | 163 | 302 | 80 | 180 | 79.6 |
| BVic-12 | 71 | 143 | 40 | 63 | 79.6 |
| BVic-13 | 125 | 170 | 90 | 127 | 45.7 |
| BVic-14 | 222 | 339 | 107 | 302 | 83.8 |
| BVic-15 | 74 | 160 | 40 | 63 | 85.0 |
| BVic-16 | 70 | 160 | 40 | 53 | 93.3 |
| BVic-17 | 148 | 302 | 85 | 127 | 80.8 |
| BVic-18 | 231 | 302 | 160 | 254 | 42.0 |
| BVic-19 | 120 | 160 | 80 | 135 | 41.4 |
| BVic-20 | 249 | 381 | 160 | 254 | 55.8 |
| BVic-21 | 187 | 254 | 160 | 160 | 39.9 |
| BVic-22 | 498 | 538 | 453 | 508 | 40.2 |
| BVic-23 | 411 | 604 | 302 | 381 | 45.7 |
| BVic-24 | 285 | 570 | 160 | 254 | 79.6 |
| BVic-25 | 187 | 320 | 151 | 135 | 60.3 |
| BVic-26 | 570 | 604 | 604 | 508 | 30.0 |
| BVic-27 | 190 | 269 | 160 | 160 | 35.6 |
| BVic-28 | 559 | 640 | 570 | 479 | 32.1 |
| BVic-29 | 593 | 640 | 640 | 508 | 24.7 |
| BVic-30 | 117 | 285 | 50 | 113 | 119.2 |

*^a^*Consensus GMT for each sample was calculated across all replicates, all laboratories.

*^b^*Laboratory GMT for each sample was calculated across all replicates within a laboratory.
